# Crosslinked Elastomers: Structure–Property Relationships and Stress-Optical Law

**DOI:** 10.3390/polym14010009

**Published:** 2021-12-21

**Authors:** Paul Sotta, Pierre-Antoine Albouy, Mohammad Abou Taha, Benoit Moreaux, Caroline Fayolle

**Affiliations:** 1 Laboratoire Polymères et Matériaux Avancés, CNRS, UMR 5268, Solvay, F-69192 Saint-Fons, France; aboutaha.mohammad@outlook.com; 2 Ingénierie des Matériaux Polymères, Université de Lyon, CNRS, UMR 5223, INSA Lyon, Université Lyon 1, UJM, F-69621 Villeurbanne, France; 3 Laboratoire de Physique des Solides, Université Paris-Saclay, CNRS, UMR 8502, F-91405 Orsay, France; pierre-antoine.albouy@universite-paris-saclay.fr; 4 Solvay Silica, F-69660 Collonges-au-Mont-d’Or, France; benoit.moreaux@solvay.com (B.M.); caroline.fayolle@solvay.com (C.F.)

**Keywords:** crosslinked elastomers, crosslink density, stress-optical law

## Abstract

We present a combination of independent techniques in order to characterize crosslinked elastomers. We combine well-established macroscopic methods, such as rheological and mechanical experiments and equilibrium swelling measurements, a more advanced technique such as proton multiple-quantum NMR, and a new method to measure stress-induced segmental orientation by in situ tensile X-ray scattering. All of these techniques give access to the response of the elastomer network in relation to the crosslinking of the systems. Based on entropic elasticity theory, all these quantities are related to segmental orientation effects through the so-called stress-optical law. By means of the combination of these techniques, we investigate a set of unfilled sulfur-vulcanized styrene butadiene rubber elastomers with different levels of crosslinking. We validate that the results of all methods correlate very well. The relevance of this approach is that it can be applied in any elastomer materials, including materials representative of various industrial application, without prerequisite as regards, e.g., optical transparency or simplified formulation. Moreover, the approach may be used to study reinforcement effects in filled elastomers with nanoparticles.

## 1. Introduction

In this paper, we present a combined experimental approach that allows characterizing the relationship between the response to various types of constraint and the structure of the crosslinking network in elastomer materials. Elastomers are very important polymeric materials as they exhibit unique mechanical properties. For practical applications, they generally need to be reinforced by adding solid particles or aggregates (fillers) of sub-micrometric sizes. The most commonly used reinforcing nanoparticles are carbon black or either fumed or precipitated silica [1,2,3,4,5,6,7], which brings qualitatively new mechanical behavior and drastically improves the properties of the obtained materials in terms of elastic modulus and energy at break [8]. Indeed, the modulus in the linear regime may be enhanced by more than two orders of magnitude, as illustrated in the seminal paper by Payne [9]. The reinforcement ratio commonly reaches 50 and strongly depends on temperature [8,10]. Reinforcement is a complex phenomenon and eventually involves several distinct mechanisms related with the structure and dynamics at the molecular level [8,11,12,13,14]. Local strain amplification in the elastomer matrix due to the filler volume effect, filler–filler networking, filler–rubber interactions, and long-range modification of the molecular dynamics within the elastomer matrix have been identified as the most important ones [15,16,17]. The relative quantitative importance of these various factors in various elastomer materials is frequently debated still. For these reasons, it is essential to be able to discriminate and quantify each of these factors.

Reinforcement of elastomers by nanoparticles has been often addressed and characterized in terms of the overall mechanical response of the material only [18,19]. Then the behavior of the reinforced material can be compared to that of the unreinforced one, based on the hypothesis that the molecular structure of the unreinforced elastomer reflects that of the matrix in the reinforced material in an exact way. Another approach consists of characterizing the behavior of the elastomer matrix within a reinforced material in situ, in a selective way. In such an approach, techniques or combinations of techniques that may enable characterizing the behavior of the elastomer matrix in both pure (unfilled) and filled materials must be used. The overall response of the reinforced material may then be compared or paralleled with the local response of the elastomer matrix, and this comparison may allow discriminating reinforcement mechanisms and contributions originating from the response of the matrix (strain amplification, local strain, or stress concentration), and those originating from the filler network [20,21].

In this paper, we present a combination of various experimental techniques which gives precise insight on the molecular characterization of elastomers. The approach was already introduced in natural rubber (NR) elastomer materials [22]. Here, we extend it to a series of SBR materials to assess the generality of the approach. The aim of this article is to introduce our approach, which combines multiple-quantum (MQ) proton NMR, measurements of the torque during crosslinking, equilibrium swelling experiments, mechanical experiments, and amorphous phase anisotropy measurements under strain by wide angle X-ray diffraction. While some among these techniques have been used routinely for decades, such as rheometry, swelling, and mechanical measurements, or more recently, e.g., MQ-NMR, orientation measurements by X-ray diffraction is much more recent [22,23]. Besides, combining all these techniques to obtain quantitative structure–property relationships in elastomers is quite innovative. All these techniques give access to segmental orientation effects and/or chain elastic response, measured in different ways. Therefore, all techniques give results which can be related essentially to one main parameter, which is the average crosslink density (or, equivalently, the average length of network chains). Here, we describe in detail the various experimental techniques and show how their results are correlated to each other. Indeed, one purpose of this paper is to demonstrate the concordance of all these measurements. Specifically, we report and discuss the correlation between amorphous phase anisotropy measurements by X-ray scattering and the results of other techniques. Studying pure elastomer matrices is a prerequisite to study reinforced materials, because it is the way in which the correlations between the various measured quantities will be affected in reinforced materials which shall give some hints on reinforcement mechanisms. As mentioned above, one ultimate interest of the approach lies in reinforced materials. It has been shown that transposing such a combination of measurements to reinforced materials indeed gives some new pieces of information on reinforcement mechanisms, by comparing to pure (non-reinforced) materials with similar elastomer matrices [24,25].

In unfilled elastomers, the detailed mechanical behavior is essentially related to the complex topology of the crosslink network and the conformation of chains within this network. Besides mechanical measurements, many different experimental approaches have been used in order to study elastomer networks. These include techniques to measure segmental orientation, such as optical birefringence [26,27], fluorescence polarization [28], infrared dichroism [29], and ${}^{2}$H NMR [30,31,32,33,34,35,36]. Small angle scattering techniques have been used as well [37,38,39,40,41,42].

Time-domain NMR techniques such as proton MQ experiments, which can be applied on low resolution, low-field spectrometers, have been developed to characterize elastomer networks [43,44]. These measurements are based on the quantitative determination of partially averaged residual dipolar couplings between protons, under the effect of the induced local order due to the orientation dependence of the chain segments constrained by crosslinks and entanglements. These residual dipolar couplings are the responsibility of a build-up signal dominated by spin-pair double-quantum (DQ) coherences. MQ-NMR is one of the most quantitative and reliable methods for the measurement of residual dipolar couplings, and thus to characterize elastomer networks. In MQ-NMR, the temperature-independent effects of the network structure can be quantitatively separated from the temperature-dependent segmental dynamics, just by proper signal normalization. Moreover, by a suitable data analysis, it is possible to access the whole distribution of residual dipolar couplings and, from them, calculate the average crosslink density of networks and its distribution (heterogeneities) in relation to the physico-chemical characteristics of the elastomer network [45,46]. Note also that time-domain proton NMR performed on samples stretched in situ was performed [47].

Equilibrium swelling experiments in a good solvent have also been widely used to characterize elastomer network structures in rubber science and technology. The classical Flory–Rehner equation [48,49,50], based on the elastic response of polymer chains to the osmotic stress of the solvent, directly relates the rubber volume fraction at swelling equilibrium to the average molecular weight between crosslinks. Thus, the average molecular weight between crosslinks can be determined in a simple way, even though experiments must be conducted and analyzed very precisely [51]. Different expressions are available, according to whether the swelling is assumed to be described by an affine or phantom network model. Note that these determinations are quite sensitive to the precise value of the Flory–Huggins interaction parameter χ [52,53] which describes elastomer–solvent interactions.

X-ray diffraction has been used for decades to characterize strain-induced crystallization (SIC) in NR [54,55,56,57,58,59,60,61,62,63,64,65,66,67]. SIC is generally considered to be responsible for the high mechanical and ultimate performances of NR. In addition to characterizing the onset, equilibrium value, and kinetics of SIC, it has been shown recently that quantitative measurements of the amorphous phase orientation can be obtained by analysis of X-ray diffraction patterns obtained in samples stretched in situ [68]. Indeed, under uniaxial stretching, an anisotropy is observed in the amorphous scattering, which can be related to the average orientation of network chain segments in the amorphous phase.

Recently, this combination of techniques has been applied to a set of natural rubber (NR) elastomers with different levels of crosslinking [22]. Here, we demonstrate that the same combination of techniques may be applied in another type of elastomer matrix, namely well-defined, sulfur vulcanized styrene butadiene rubber (SBR) with various crosslink densities, which, in contrast to NR, does not crystallize under strain due to the non-regular conformation of its chain backbone. In addition to the previous set of combined characterization methods, we show here that the rheological response measured in real time during the curing process also correlates quantitatively to the crosslink density and therefore can be used to characterize these materials.

The paper is organized as follows. In Section 2, we give some general, basic background on the analysis of the results of the various techniques which are used, based on basic rubber elasticity theory. In Section 3, we describe in detail the samples, the experimental techniques which have been used, and the way in which results have been analyzed. Results are shown and discussed in Section 4.

## 2. Basis of the Approach

The approach relies on the so-called stress-optical law (Equation (1)), which relates the stress to molecular orientation at the scale of chain segments [69]:

(1) $$\sigma_{ij}=\frac{3k_{B}T}{b^{3}}\langle u_{i}u_{j}-\frac{\delta_{ij}}{3}\rangle ,$$

where *T* is the temperature, $k_{B}=1.38\times 10^{-23}$ J K${}^{-1}$ the Boltzmann constant, $\sigma_{ij}$ are the components of the true stress tensor, $u_{i}$ is the *i*th component of the unit vector parallel to a polymer chain segment, and $b^{3}$ is the volume of a statistical segment. $\delta_{ij}$ is the Kronecker symbol ($\delta_{ij}=1$ if i=j, =0 otherwise). This relationship is the core of the entropic elasticity theory. In fact, its importance generality goes well beyond the particular case of crosslinked elastomer networks and is at the basis of the whole viscoelastic behavior of polymers. Besides, on a general basis, it should be valid even in the non-linear regime [69].

Brackets in Equation (1) denote statistical averaging over the ensemble of chain segments in the system. It is important to realize that entropic elasticity is based on strong hypotheses on the local dynamics. Two distinct time scales should be clearly separated in the system [70]. A permanent or slowly relaxing network of topological constraints should exist in the system to insure elastic response, while small scale segmental reorientations in between constraints should be fast enough to insure full time averaging on experimental time scale. The related elastic modulus, corresponding to a stored elastic energy of order $k_{B}T$ per chain, that is, over a volume of a few nm${}^{3}$, is then of order a fraction of to a few megapascals. Entropic elasticity is thus fundamentally distinct from solid-state elasticity, in which the mechanical response is driven by intermolecular forces and the elastic modulus is in the range of one to a few gigapascals.

For the particular case of uniaxial extension, Equation (1) resumes to Equation (2):

(2) $$\sigma =\frac{3k_{B}T}{b^{3}}\langle P_{2}\left(\cos\theta \right)\rangle ,$$

where σ is the true tensile stress and $\langle P_{2}\left(\cos\theta \right)\rangle =\langle (3\cos^{2}\theta -1)/2\rangle$, with θ the angle between a chain segment and the tensile direction. In the same way as in Equation (1), brackets denote an ensemble average over the ensemble of chain segments in the elastomer.

Based on standard polymer chain statistics in the Gaussian regime, the local segmental orientation parameter $\langle P_{2}\left(\cos\theta \right)\rangle$ may be related to the elongation ratio $\lambda =L/L_{0}$ as in Equation (3):

(3) $$\langle P_{2}\left(\cos\theta \right)\rangle =b^{3}\nu \rho_{r}\psi \left(\lambda^{2}-\lambda^{-1}\right),$$

where ν is the crosslink density (in kg${}^{-1}$), $\rho_{r}$ the rubber density (in kg·m${}^{-3}$), $\lambda =l/l_{0}$ ($l_{0}$ (resp. *l*) is the initial (resp. elongated) length of the sample) is the elongation ratio, and ψ is a factor which depends on the way in which crosslink positions move and fluctuate under the applied strain. Under the hypothesis of affine deformation ψ=1, while for a phantom network model, ψ=(f−2)/f, where *f* is the network functionality [71], taken here to be typically f=4, which leads to ψ=1/2.

It is clear that changes in the way in which crosslinks or, more generally, topological constraints, are accounted for may affect the segmental orientation and may potentially invalidate Equation (3) [72]. Whether the stress-optical law in elastomers remains strictly valid or not is a non-fully settled issue yet, as far as we know. For instance, both the classical constrained junction model and the diffuse-constraint theory predict that the strict proportionality is not maintained [73,74]. On the other hand, it is still valid in the slip-link model [75]. Recent measurements show deviations which do not seem to be fully accounted for by existing models [76].

### 2.1. Mechanical Experiments

By combining the above Equations (2) and (3), the linear regime of rubber elasticity in uniaxial stretching is characterized by the following Equation (4), between the true stress σ and the elongation ratio λ [71,77,78,79]:

(4) $$\sigma =k_{B}T\nu \rho_{r}\psi \left(\lambda^{2}-\lambda^{-1}\right).$$

The crosslink density ν is related to the average chain molecular weight $M_{c}$ between consecutive crosslinks by $\nu =1/2M_{c}$ (assuming tetra-functional crosslinks). The factor $G=k_{B}T\nu \rho_{r}\psi$ is the shear modulus. As already mentioned, Equation (4) is based on the assumption that the system has fast local dynamics and is subject to a given set of permanent constraints, namely chemical crosslinks and other topological constraints generically denoted as trapped entanglements. Thus, the effective crosslink density ν should be understood here as including both chemical crosslinks and trapped entanglements which have a permanent elastic effect over the time scale of the measurement.

More detailed models have been derived to account for various complex aspects of network topology and of the effect of deformation on local constraints exerted on chains, but the main physical ingredients remain similarly based on entropic elasticity [73,80,81,82,83,84,85,86,87,88]. Moreover, the linear relationships described by Equations (2) and (3) may be valid up to relatively large extension degrees. It follows that Equation (4) should also be valid up to large extension. However, this is often not strictly observed in practice and the behavior is rather described by the classical Mooney–Rivlin Equation (5) [89,90]:

(5) $$\sigma =\left(C_{1}+\frac{C_{2}}{\lambda }\right)\left(\lambda^{2}-\lambda^{-1}\right),$$

which has been interpreted as due to the release of a fraction of the entanglements under the effect of the strain and/or the complex interplay between the strain and the local constraints exerted on network chains [73].

Nevertheless, it follows from Equations (3) and (4) that, in a given type of elastomer material, the primary material parameter which drives the mechanical response in the small/medium strain amplitude is the average crosslink density. Ultimate properties, such as resistance to tear and energy at break, depend on the crosslink density as well, but in a more complex yet poorly understood way. Ultimate properties also depend crucially on more involved details of the network topology, such as the presence of defects and the homogeneity of the network in terms of crosslink density.

In what follows, we shall review our multi-scale approach combining measurements of various quantities related to the material response at various scales, all measurements involving the crosslink density as the main material parameter. On one hand, we shall use various methods to estimate/measure the crosslink density. On the other hand, we shall investigate the response of the material to uniaxial stretching experiments in which both the mechanical response and the orientation are measured in real time.

### 2.2. Measurement of the Crosslink Density by Time-Domain Proton NMR

Measurements of the crosslink density by time-domain proton NMR spectroscopy heavily relies on the dynamical assumptions detailed above. The measured quantity is the residual tensorial interaction $D_{res}$ which originates from incomplete motional averaging of chain segments fluctuating rapidly between topological constraints, such as crosslinks or chain entanglements. Local reorientation motions are anisotropic due to topological constraints, even though the system is overall isotropic in the relaxed state. The measured quantity is then the nonzero time average within a given network strand of the second order Legendre polynomial $P_{2}\left(\cos\theta \right)$, in which θ is the time-dependent angle between the local chain direction (segmental orientation) and a reference direction.

The overall measured quantity is then the average over all network chains, denoted $S_{b}$, of this local time average of the polymer backbone orientation, related to the average number of statistical segments *N* or, equivalently, the molecular mass $M_{c}$, between constraints and to the statistical segment length *b*. This leads to Equation (6):

(6) $$S_{b}\propto \frac{3}{5}\frac{R^{2}}{b^{2}N^{2}}\propto \frac{1}{N}\propto \frac{1}{M_{c}}\propto \nu ,$$

in which $R^{2}\approx b^{2}N$ is the average squared end-to-end distance of a network strand. Since the proton dipolar coupling, which is the NMR observable, depends on molecular orientation, the nonzero dynamic orientation of the polymer backbone $S_{b}$ is detected in NMR because it gives a nonzero residual dipolar coupling $D_{res}$. $S_{b}$ is calculated from the experimental average residual dipolar coupling constant $D_{res}$, by comparison with its static counterpart, $D_{static}$ as expressed in Equation (7) (*k* is a correction factor <1 accounting for the spin arrangement and motions within a statistical segment) [44]:

(7) $$S_{b}=k\frac{D_{res}}{D_{static}}$$

According to Equations (6) and (7), $D_{res}$ is inversely proportional to the average molecular weight of network chains between crosslinks $M_{c}$ or, equivalently, proportional to the crosslink density ν. Entanglements also contribute to the NMR signal. Assuming a constant entanglement density and simple additivity of entanglement and crosslink densities, we may write $D_{res}\propto 1/M_{c}+1/M_{e}$, with $M_{e}$ the entanglement molecular weight. Note, however, that this assumption is certainly oversimplified. In vulcanized samples, the density of trapped entanglements may itself depend on the crosslink density. In fact, it has been suggested theoretically that, at lower crosslink densities, the linear variation of $D_{res}$ toward a finite ordinate value proportional to $1/M_{e}$ may change to a square-root behavior ∼$1/\sqrt{M_{c}M_{e}}$ in the very high temperature limit [91]. This argument is based on the orientational averaging behavior of network chains within the tube arising from entanglement constraints. The resulting decay of $D_{res}$ towards zero is, however, generally not found in experiments as the timescale of large-scale chain motions within the tube is generally much longer than the NMR experimental timescale at relevant temperatures, thus preserving the effective linear decay towards $1/M_{e}$.

### 2.3. Measurement of the Crosslink Density by Equilibrium Swelling Experiments

Equilibrium swelling experiments allow determining the average molecular weight between crosslinks $M_{c}$ (in g·mol${}^{-1}$) (or, equivalently, the crosslink density $\nu_{sw}=1/2M_{c}$) by means of the thermodynamic description based on the Flory–Rehner theory [48,49,50] of swollen networks. For a network immersed in a solvent, the network density at swelling equilibrium is based on the balance between the elastic term of extended network chains and the free energy of mixing. The mixing term is related to the interactions between the polymer and the swelling solvent and is commonly computed by the Flory–Huggins solution theory [52,53]. On the other hand, the elastic term depends on the model used to describe the network [71,79,92]. Two different models of network deformation are mainly used to describe the behavior of crosslinked rubbers: (i) the affine deformation model, which states that the deformation applied to crosslink positions is the same as the macroscopic deformation imposed to the overall network, and (ii) the phantom model, which assumes that the positions of the crosslinks are not fixed and can fluctuate. For the affine deformation model, the classical Flory–Rehner theory, which relates the rubber volume fraction $\phi_{r}$ at swelling equilibrium (or, equivalently, the degree of swelling $Q=V/V_{0}=1/\phi_{r}$) to $M_{c}$, is expressed as in Equation (8):

(8) $$\ln\left(1-\phi_{r}\right)+\phi_{r}+\chi \phi_{r}^{2}=-\frac{\rho_{r}}{M_{c}}V_{s}\left(\phi_{r}^{1/3}-\frac{2\phi_{r}}{f}\right),$$

where $\rho_{r}$ is the rubber density, $V_{s}$ the solvent molar volume, χ the Flory–Huggins polymer–solvent interaction parameter, *f* the crosslink functionality. On the other hand, for the phantom model, the formula reads as in Equation (9):

(9) $$\ln\left(1-\phi_{r}\right)+\phi_{r}+\chi \phi_{r}^{2}=-\frac{\rho_{r}}{M_{c}}V_{s}\left(1-\frac{2}{f}\right)\phi_{r}^{1/3}.$$

It is generally considered that the real behavior of swollen elastomer networks is better described by the phantom expression. The details of the method and the differences between the models were discussed in detail by Valentín et al. in [51].

### 2.4. X-ray Scattering

In a stretched elastomer network, it has been observed that the amorphous scattering halo, which comes from liquid-like monomer–monomer or chain–chain short range interferences, becomes anisotropic under elongation, with more intensity in directions perpendicular to the stretching direction [56,93]. Analyzing the azimuth dependence of the scattered intensity enables extracting an anisotropy parameter ${\langle P_{2}\rangle }_{X}$, where the *X* suffix indicates that the quantity reflects the anisotropy of the X scattering pattern. The amorphous scattering predominantly comes from inter-chain atom–atom correlation at the monomer scale, and it is difficult to relate it quantitatively to the average segmental orientation parameter $\langle P_{2}\rangle$ [67,94]. However, based on symmetry consideration, it may be stated that ${\langle P_{2}\rangle }_{X}$ is proportional to the orientation parameter $\langle P_{2}\rangle$ introduced in Equations (2) and (3) (with a negative proportionality factor as the scattering is enhanced in the direction perpendicular to the tensile direction).

From standard elasticity theory, according to Equation (3)$,{\langle P_{2}\rangle }_{X}$ can be written as in Equation (10)

(10) $${\langle P_{2}\rangle }_{X}=\frac{K\psi }{5N}\left(\lambda^{2}-\lambda^{-1}\right),$$

where *K* is a (negative) factor related to the local structure of the amorphous phase. An estimated value was given by Mitchell in an early publication [56], but it is presently considered as unknown in view of the uncertainties involved. Thus, the average orientation induced upon stretching, as introduced in Equations (2) and (3), can thus be measured by X-ray scattering to within a proportionality factor. However, this factor is a priori independent of strain and crosslink density so that reliable relative comparisons may be performed.

Note again that the average orientational order parameter $\langle P_{2}\rangle$, as involved in Equations (2) and (3), or, equivalently, ${\langle P_{2}\rangle }_{X}$ in Equation (10) measured by wide angle X-ray scattering, should be clearly distinguished from the average *dynamic* order parameter $S_{b}$ as measured by NMR. Even though both quantities are of course related to each other and vary in the same way as a function of the crosslink density, they are not of the same nature. While $\langle P_{2}\rangle$ is an ensemble average which expresses the response of the material to an applied strain and is zero in the relaxed state, $S_{b}$ reflects a local time average over fast motions inside a network strand. $S_{b}$ is related to the local structure of the network and is measured in the relaxed state. At high temperature (so that local reorientational motions are fast), both quantities are functions of the crosslink density through arguments based on chain statistics in rubber elasticity theory.

## 3. Materials and Methods

### 3.1. Samples

The investigated elastomers are Styrene butadiene rubber materials (SBR, oil extended grade SBR4526-2HM from LANXESS, vinyl content 45%, styrene content 26%) crosslinked to various degrees by adjusting the amounts of crosslinking agents. Samples were mixed and sulfur vulcanized following standard procedures. Curing agents (sulfur (S), from Rhein Chemie) and accelerators ((N-cyclohexyl-2-benzothiazole sulfenamide, or CBS, and Diphenyl Guanidine, or DPG, with a constant mass ratio CBS/DPG = 1.33, both from Rhein Chemie) were added on an open roll mill at low temperature (*T* = 50 ${}^{{}^{\circ}}$C, friction 1.1) to avoid premature crosslinking. Samples were formulated with 4 different sulfur amounts (from 0.4 to 2.2 phr) and for each sulfur amount, 3 different accelerator/sulfur ratios were chosen, as reported in Table 1. A pure SBR matrix with no curing agents was also measured as a reference. Besides ingredients reported in Table 1, the formulations contain 137.5 g of oil-extended SBR, 2.5 g of ZnO (Rhein Chemie), 2 g of stearic acid (pristerene 4963 from Croda), and 1.9 g of *N*-(1,3-dimethylbutyl)-*N*’-phenyl-p-phenylenediamine (6PPD, vulkanox 4020/LG from LANXESS). The glass transition temperature given by the maximum of the loss modulus $E^{\prime \prime }$ measured in DMA at 1 Hz is about −20 ${}^{{}^{\circ}}$C.

**Figure 1:**
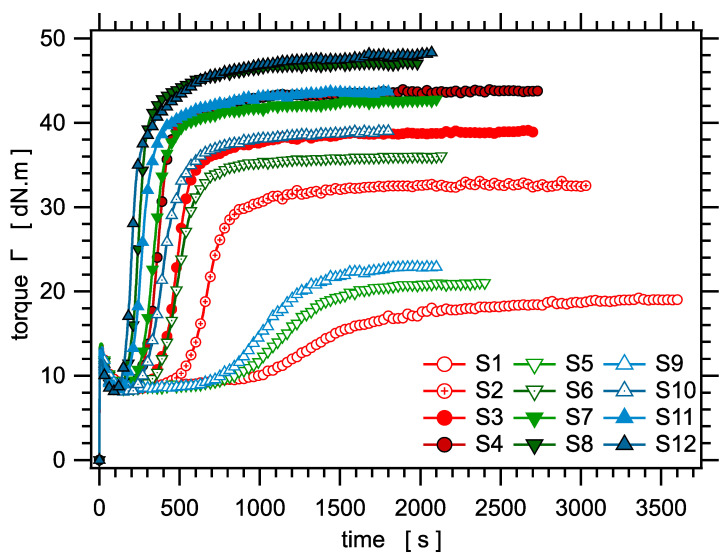
The rheological torque measured during curing for the series of crosslinked samples.

Rheological measurements during curing were performed with a Monsanto R100 Oscillating Disc Rheometer at 160 ${}^{{}^{\circ}}$C. The torque values measured during curing for all samples in the series are shown in Figure 1. The torque difference $\Delta \Gamma =\Gamma_{max}-\Gamma_{min}$ is recorded, where $\Gamma_{min}\approx 8.4$ dN·m is the torque measured in the rheometer prior to crosslinking and $\Gamma_{max}$ is the maximum torque at the optimum of crosslinking. While $\Gamma_{min}$ is related to the viscosity of the uncrosslinked polymer, ΔΓ should give the shear modulus of the crosslinked material, which is proportional to the crosslink density: $G\propto \nu \propto 1/2M_{c}$.

Equilibrium swelling in a good solvent is a classical method to determine the average molecular weight between crosslinks $M_{c}$ (or, equivalently, the crosslink density $\nu =1/2M_{c}$). The Flory–Rehner Equation (9) is generally used to relate the rubber volume fraction at swelling equilibrium $\varphi_{r}$ (or, equivalently, the degree of swelling $Q=V/V_{0}=1/\varphi_{r}$) to $M_{c}$, using the Phantom network hypothesis. Considering the crosslink density $\nu =2/M_{c}$ (for tetrafunctional crosslinks, that is f=4) and considering that a fraction of effective crosslinks (density $\nu_{0}$, corresponding to trapped entanglements) which are not active in swelling experiments are detected by NMR, Equation (9) can be rewritten as in Equation (11):

(11) $$\frac{1}{2M_{c}}=\nu =\nu_{0}-\frac{1}{\rho_{r}V_{s}}\frac{1}{{\varphi_{r}}^{1/3}}\left(\ln\left(1-\varphi_{r}\right)+\varphi_{r}+\chi {\varphi_{r}}^{2}\right).$$

Equilibrium swelling experiments were performed at room temperature by immersion in xylene. Three pieces of each sample (discs of 8 mm diameter and 2 mm thickness) were weighted initially, then swollen up to equilibrium during 72 h. The solvent was renewed once after 24 h. Samples were weighted immediately after removing from the solvent and then dried under vacuum at 40 ${}^{{}^{\circ}}$C for 24 h before being weighted again.

The following parameter values were used: $V_{s}=106.2$ cm${}^{3}$ mol${}^{-1}$, $\rho_{r}=1.087$ g.cm${}^{-3}$ (this value was verified with a pycnometer), and χ≈0.2 can be roughly estimated from the Hildebrand’s solubility parameters by the group contribution method [95] as, to our knowledge, there is no reported data for this parameter. The φ dependence of χ was not taken into account. This estimated value is lower than for the SBR–toluene pair, which is χ=0.413, which indicates that xylene is a better solvent for SBR than toluene. Measurement results for the swelling degree *Q* are reported in Table 2.

### 3.2. Time-Domain Proton DQ NMR

Proton MQ-NMR experiments were carried out at 343 K (that is, well above $T_{g}$) on a Bruker minispec mq20 spectrometer operating at 0.5 Tesla with $90^{{}^{\circ}}$ pulses of order 2 μs and a dead time of 15 μs. Well established procedures were used to obtain and analyze the normalized proton double quantum (DQ) signals in order to obtain the distribution of crosslink densities in all studied samples [44].

Two distinct signals are measured simultaneously as a function of the double quantum evolution time τ, the double quantum signal $I_{DQ}\left(\tau \right)$, and the so-called reference signal $I_{Ref}\left(\tau \right)$. The total signal $I_{tot}\left(\tau \right)=I_{Ref}\left(\tau \right)+I_{DQ}{(}_{t}au)$ contains only relaxation terms associated to the local segmental dynamics. However, it contains also the contributions of so-called defects, that is, the sol (uncrosslinked, including the oil contribution) fraction and dangling chains. This contribution $I_{def}$ is characterized by long relaxation times relative to the network contribution. The defect contribution $I_{def}$ has to be subtracted and then the normalized DQ signal $I_{NDQ}$ is computed by point-by-point normalization as in Equation (12)

(12) $$I_{NDQ}\left(\tau \right)=\frac{I_{DQ}\left(\tau \right)}{I_{tot}\left(\tau \right)-I_{def}\left(\tau \right)}.$$

**Figure 2:**
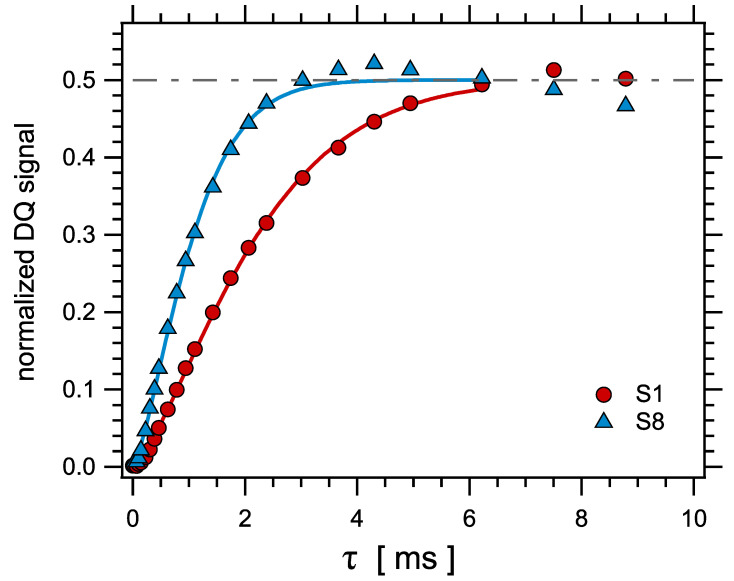
Representative normalized DQ curves obtained in samples S1 and S8. Symbols are experimental data, curves are fits with Equation (13).

In this way, in the fast motion regime, the signal $I_{NDQ}\left(\tau \right)$ contains only the contribution of residual interactions, related to the network structure. The general shape of this signal is a function which increases from zero at τ=0 up to a plateau at 0.5 at long times. Representative examples of obtained normalized DQ curves are shown in Figure 2.

For NR matrices, normalized DQ curves may be fitted with a Gaussian function and a calibration factor has been evaluated by numerical simulations to relate the NMR measured quantity $D_{res}$ to the actual molecular mass between junctions [43]. In SBR matrices, the shape of the normalized DQ curves is different and can hardly be fitted with a Gaussian function, essentially because the DQ signal combines the responses from protons located at very different sites in the chain, which then should have quite different values of the residual dipolar coupling. The normalized DQ curves were fitted up to $I_{NDQ}\approx 0.48$ with an empirical function of the form of Equation (13)

(13) $$I_{NDQ}\left(\tau \right)=\frac{1}{2}\left[1-\exp\left(-{\left(2\pi D_{res}\tau \right)}^{P_{0}}\right)\right].$$

Representative examples of such fits are shown in Figure 2.

### 3.3. Stress–Strain Curves and In Situ Wide Angle X Scattering

**Figure 3:**
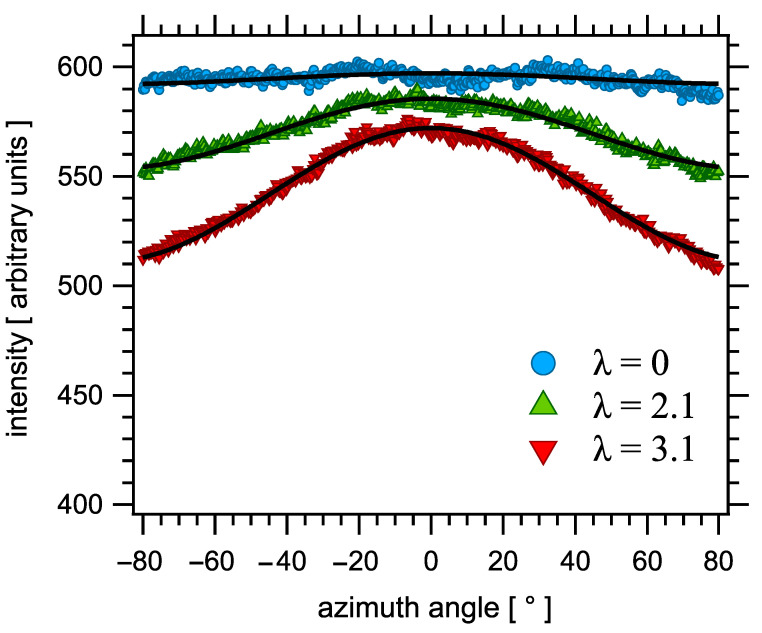
Representative integrated X intensity curves measured in sample S4 as a function of the azimuth angle φ, at various elongation ratios λ. Here, φ=0 is perpendicular to the tensile direction. Symbols are measurements, curves are the corresponding fits with the expression $A+B\cos^{2}\varphi$, from which the orientation parameter ${\langle P_{2}\rangle }_{X}$ is deduced. Data have been arbitrarily shifted vertically for better readability.

Average chain segment orientation under tensile strain was measured at 298 K with a homemade uniaxial stretching device mounted on a rotating anode X-ray generator, described in detail elsewhere [22,68]. Traction is symmetric in such a way that nearly the same zone in the sample is measured throughout the tensile test. The elongation ratio $\lambda =l/l_{0}$ at X-ray beam spot is measured simultaneously both with an optical camera and using the variation of sample thickness measured through the variation of X-ray absorption. The tensile force *F* is measured with a calibrated load cell. The true stress is defined as $F\lambda /s_{0}$, $s_{0}=6$ mm${}^{2}$ being the initial section of the samples. Two-dimensional scattering patterns were recorded in samples stretched in situ as a function of λ and the anisotropic intensity in the amorphous halo (with more (resp. less) intensity in direction perpendicular (resp. parallel) to the stretching direction) was fitted as a function of the azimuthal angle φ with the expression $A+B\cos^{2}\varphi$ (where *A* is corrected for air scattering). The anisotropy of the scattered intensity may then be characterized by a parameter ${\langle P_{2}\rangle }_{X}=2B/\left(15A+10B\right)$, which, as quoted above, is proportional to the orientation order parameter $\langle P_{2}\left(\cos\theta \right)\rangle$ [22,68]. Representative intensity curves are shown in Figure 3.

## 4. Results

### 4.1. Crosslink Densities Measured by NMR

**Figure 4:**
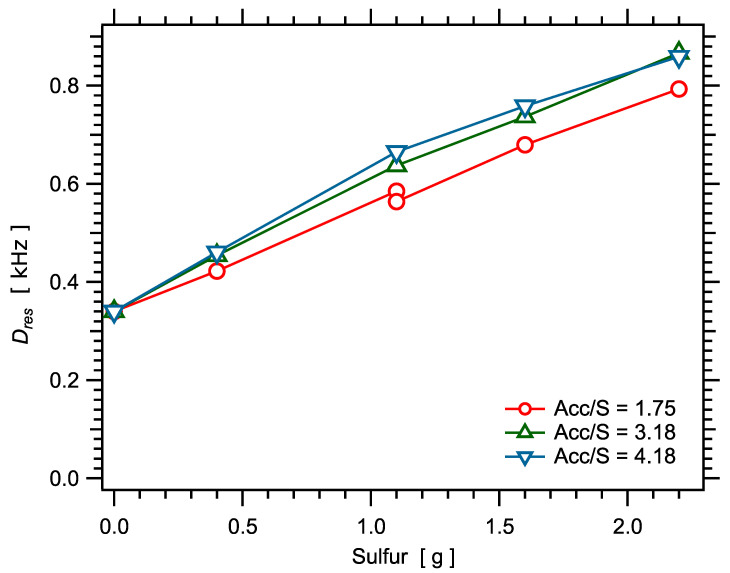
Crosslink density as a function of the sulfur amount in the series of SBR samples.

**Figure 5:**
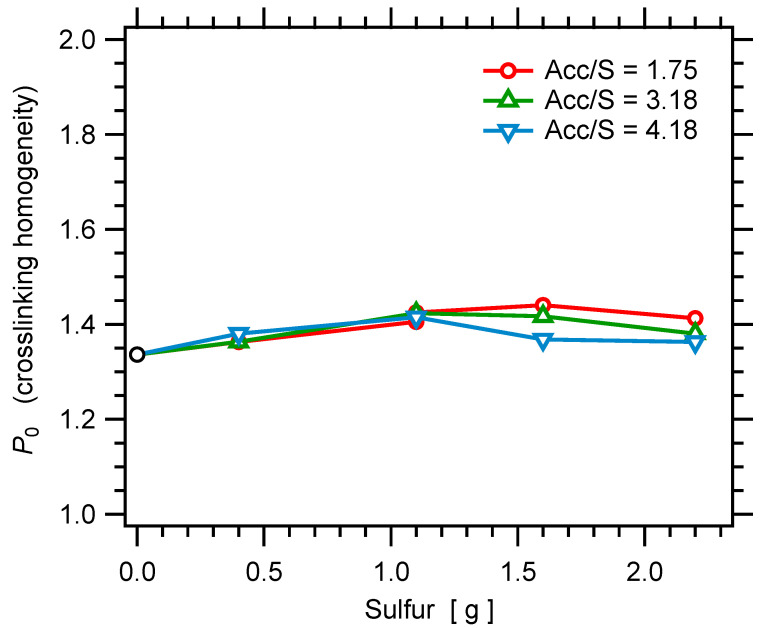
Parameter $P_{0}$, related to the homogeneity of the crosslink density, as a function of the sulfur amount in the series of SBR samples.

Results for the measured NMR quantity $D_{res}$ (proportional to the crosslink density) are shown in Figure 4. The nonzero $D_{res}$ value measured for zero sulfur corresponds to the contribution of entanglements. Then, for each value of the Acc/S ratio, $D_{res}$ shows a linear variation with the sulfur amount, which indicates that the same sulfur bridges (same average number of sulfur atoms per crosslink) are formed [96]. This is expected since the ratio Acc/S has to be changed in a quite large range to strongly affect the average number of sulfur per crosslink [96]. Nevertheless, the slope tends to increase as the ratio Acc/S increases, which is in qualitative agreement with previously published studies [96]. The values of the fitting parameter $P_{0}$ (which is interpreted as being related to the homogeneity of the crosslink density) show no significant variation in the whole series of unfilled samples, as shown in Figure 5. Equivalently, when plotted as a function of the rescaled time $\tau_{DQ}/D_{res}$, all DQ curves fall on a single master curve. This indicates that all samples have the same degree of crosslinking homogeneity.

### 4.2. Equilibrium Swelling

**Figure 6:**
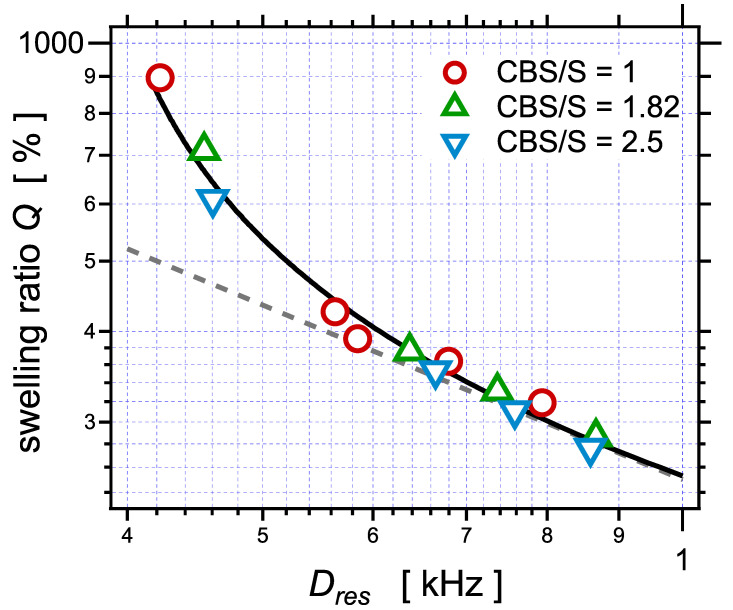
The equilibrium swelling ratio *Q* (in vol%) as a function of the NMR parameter $D_{res}$, for the series of samples with different amounts of sulfur and different sulfur/accelerator ratios. The plain curve is a fit of the full set of data with the Flory–Rehner equation (Equation (11)), taking the assumption of phantom network, as described in the text.

Figure 6 shows the equilibrium swelling ratio $Q=100/\varphi_{r}$ (in vol% of the swollen volume over the volume of the elastomer network) as a function of the parameter $D_{res}$ determined by NMR. Considering that $D_{res}$ is proportional to the crosslink density $D_{res}\propto \nu \propto 1/M_{c}$, the full set of swelling data was fitted with an equation similar to the Flory–Rehner Equation (11). An offset $D_{res}^{\left(0\right)}$ related to the contribution of entanglements and corresponding to the nonzero ordinate in the curves in Figure 4 was considered, leading to Equation (14):

(14) $$D_{res}=D_{res}^{\left(0\right)}-\frac{P_{1}}{{\varphi_{r}}^{1/3}}\left(\ln\left(1-\varphi_{r}\right)+\varphi_{r}+\chi {\varphi_{r}}^{2}\right).$$

The obtained values of the fitting parameters are $D_{res}^{\left(0\right)}=0.36$, χ=0.18, and $P_{1}=5.98$. This fit enables estimating the proportionality factor between $D_{res}$ and $M_{c}$ in a quantitative way, as given by Equation (15):

(15) $$D_{res}-D_{res}^{\left(0\right)}\approx \frac{345}{M_{c}},$$

with $D_{res}$ in Hz and $M_{c}$ in kg/mol. Moreover, shown in Figure 6 is the scaling law $Q\propto D_{res}^{-4/5}$ adjusted to the experimental points in the limit of high crosslink densities. This follows from the scaling consideration that, at swelling equilibrium, the swollen gel consists in a packing of volumes $R_{F}^{3}\approx {\left(b^{2}N^{3/5}\right)}^{3}$ occupied by one network strand of length *N* segments, $R_{F}$ being the average dimension of the strand swollen in good solvent, with 3/5 the Flory exponent [92]. Within such a picture, the swelling ratio then scales as $Q\approx R_{F}^{3}/\left(b^{3}N\right)\propto N^{4/5}\propto D_{res}^{-4/5}$.

**Figure 7:**
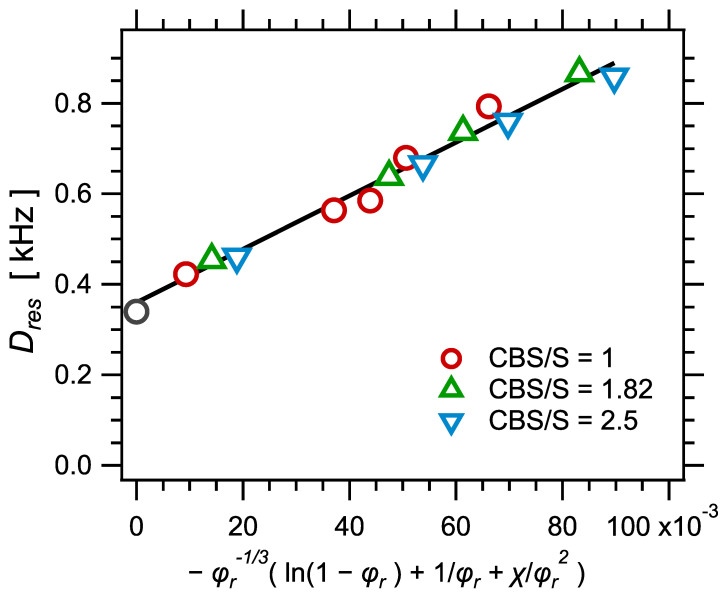
The NMR parameter $D_{res}$ as a function of the quantity $-{\varphi_{r}}^{-1/3}\left(\ln\left(1-\varphi_{r}\right)+\varphi_{r}+\chi {\varphi_{r}}^{2}\right)$, with φ=100/Q. According to the Flory–Rehner model, Equation (11), and taking the assumption of phantom network, the relationship should be linear, which is well verified. The line is the obtained best linear fit. The ordinate at origin $D_{res}^{\left(0\right)}\approx 0.35$ corresponds to the contribution of entanglements.

Another equivalent way to emphasize the good correlation between swelling and NMR measurements, and visualize the proportionality factor between $D_{res}$ and $1/M_{c}$, is to plot the NMR parameter $D_{res}$ as a function of the quantity $-{\varphi_{r}}^{-1/3}\left(\ln\left(1-\varphi_{r}\right)+\varphi_{r}+\chi {\varphi_{r}}^{2}\right)$ (with φ=100/Q). This is shown in Figure 7. Figure 7 contains the same kind of information as Figure 6. It also allows to visualize clearly the fraction of "effective" crosslinks which contribute to the NMR response and do not in equilibrium swelling measurements, associated to the ordinate $D_{res}^{\left(0\right)}$. These "effective" crosslinks may be either trapped entanglements, which are released upon swelling, or topological constraints, active on the time scale of the NMR measurements (of the order $10^{4}$ Hz) but relaxed on the much longer swelling times.

### 4.3. Rheological Response during Curing

**Figure 8:**
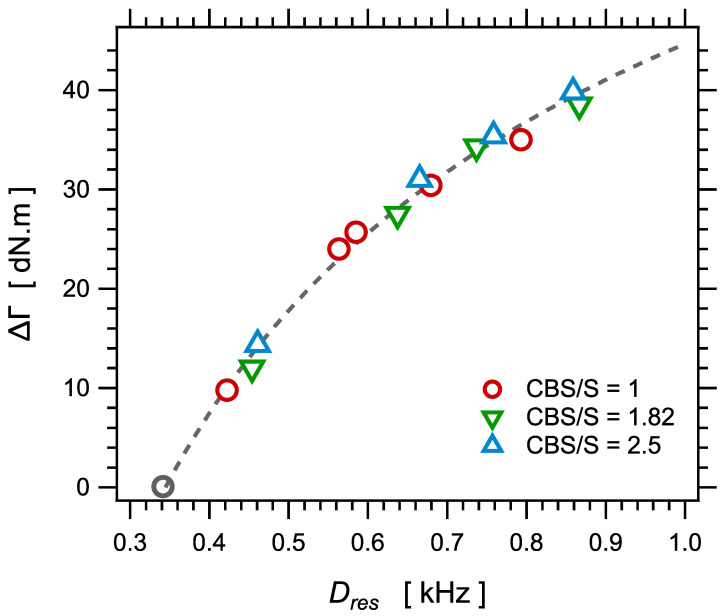
The torque increase ΔΓ during curing as a function of the NMR parameter $D_{res}$, for the series of samples with different amounts of sulfur and different sulfur/accelerator ratios. The dashed curve is a guide for the eye.

Let us examine now the correlation with torque measurements during curing. The torque increase ΔΓ during curing is plotted as a function of the NMR parameter $D_{res}$ in Figure 8. Assuming that both quantities would vary linearly with the crosslink density $\nu \propto 1/M_{c}$, the relationship between ΔΓ and $D_{res}$ should be linear. Even though both quantities clearly are strongly correlated in the sense that they nicely collapse on a master curve, the relationship is not linear. The torque difference increases slower than linear with $D_{res}$. The dashed curve in Figure 8 is only a guide for the eye. We do not have a clear explanation for this deviation with respect to linear variation.

**Figure 9:**
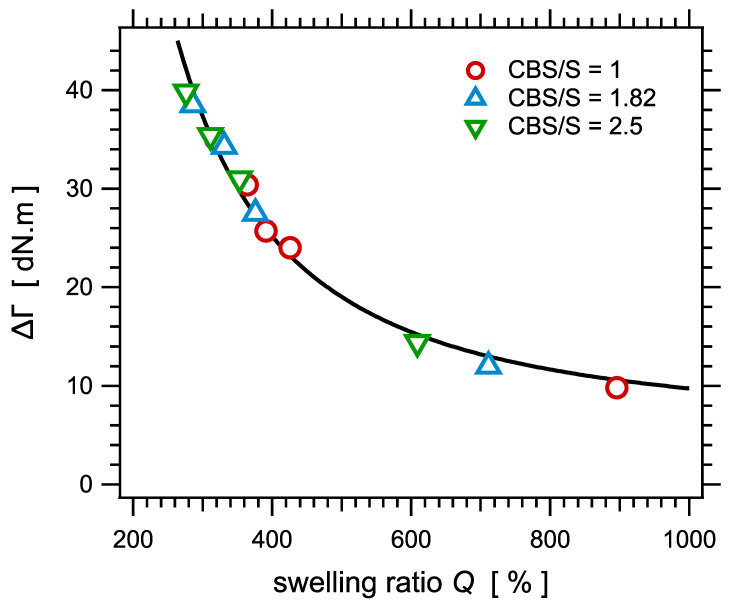
The torque increase ΔΓ during curing as a function of the equilibrium swelling ratio *Q*, for the series of samples with different amounts of sulfur and different sulfur/accelerator ratios. The curve is a fit with an equation similar to Equation (14).

The torque increase ΔΓ is plotted as a function of the swelling ratio *Q* in Figure 9. Again, the curve can be fitted with Equation (11), as shown in the figure.

### 4.4. Mechanical Response: Stress–Strain Curves

**Figure 10:**
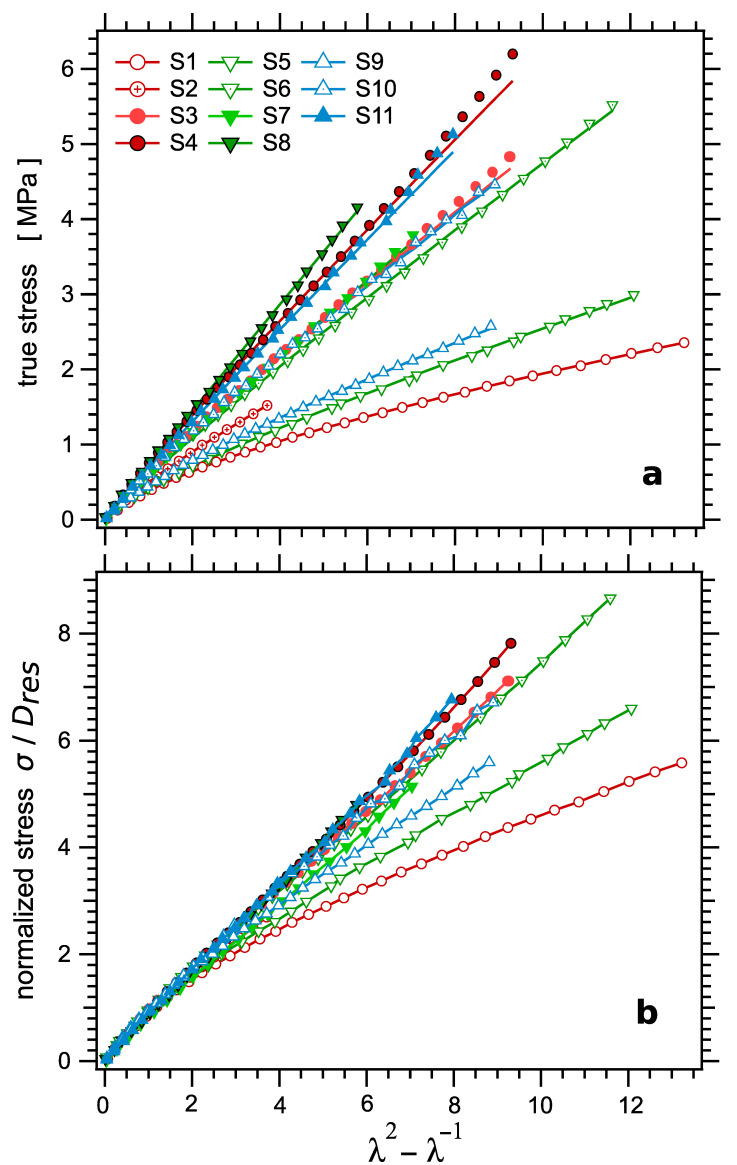
(**a**) The true stress σ as a function of the elongation parameter $\lambda^{2}-\lambda^{-1}$ for the series of crosslinked samples. Symbols are experimental points, curves are Mooney–Rivlin fits, according to Equation (16). The graph illustrates the upwards deviation affecting some of the curves at high stress. (**b**) The normalized stress $\sigma /D_{res}$ as a function of the elongation parameter $\lambda^{2}-\lambda^{-1}$ for the series of crosslinked samples. As expected, the curves superpose in the low strain regime, up to about 60% strain.

Next, the responses of the samples to uniaxial loading were investigated. The mechanical response is discussed first. The true stress curves are plotted as a function of the elongation parameter $\lambda^{2}-\lambda^{-1}$ for the whole set of samples in Figure 10a. The curves can be perfectly fitted by the classical Mooney–Rivlin Equation (16) [89,90]:

(16) $$\sigma =\left(C_{1}+\frac{C_{2}}{\lambda }\right)\left(\lambda^{2}-\lambda^{-1}\right).$$

**Figure 11:**
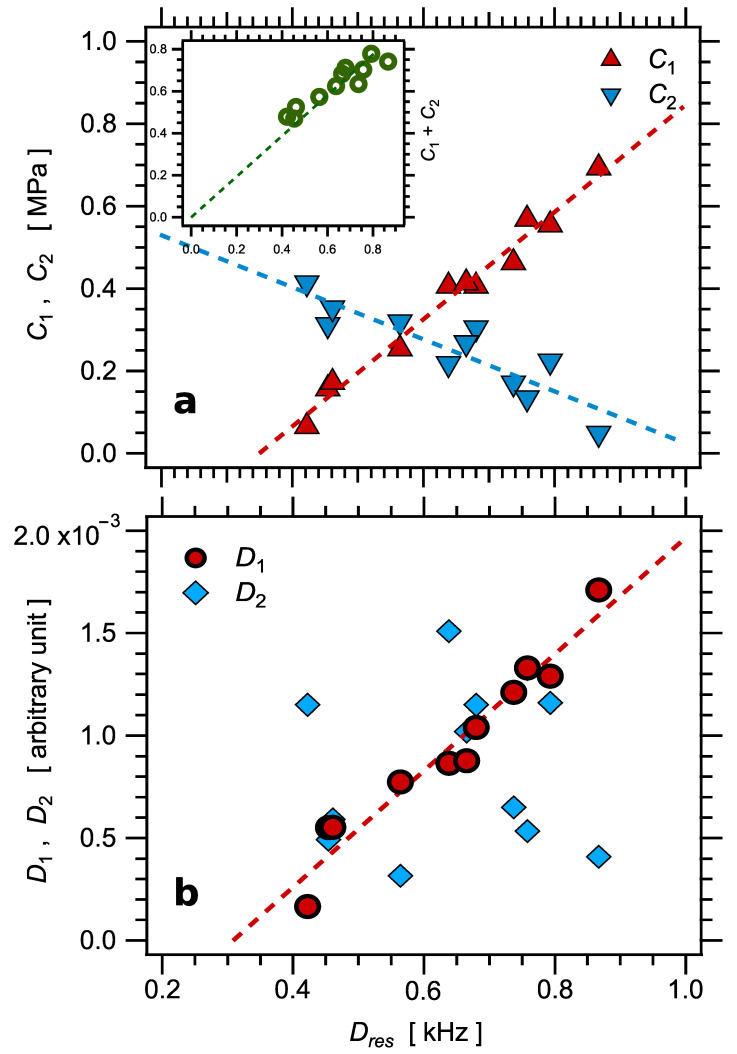
(**a**) TheMooney–Rivlin coefficients $C_{1}$ and $C_{2}$ fitted on the tensile stress–strain curves shown in Figure 10 as a function of $D_{res}$. Inset: the zero strain modulus $C_{1}+C_{2}$ as a function of $D_{res}$. (**b**) The effective Mooney–Rivlin coefficients $D_{1}$ and $D_{2}$ fitted on the $\langle P2\rangle$ curves shown in Figure 13a. Dashed lines are linear fits of corresponding data and should be considered as guides for the eye.

The fitted Mooney–Rivlin parameters $C_{1}$ and $C_{2}$ are reported in Table 3 and plotted as a function of the sulfur amount in Figure 11a. As it is often observed, the Mooney–Rivlin coefficients show a defined trend, with the ratio $C_{2}/C_{1}$ increasing quite largely as the crosslink density decreases, that is, as the zero strain modulus decreases. This trend is illustrated by dashed lines in Figure 11a. The modulus at large strain $C_{1}$ shows a linear trend as a function of $D_{res}$ with, however, a nonzero extrapolated value of order $D_{res}\approx 0.35$ kHz at $C_{1}=0$. This would correspond to the uncrosslinked material and is indeed equal to the $D_{res}$ value measured in this sample. Conversely, the zero strain modulus $C_{1}+C_{2}$ shows also a linear trend, with much more scatter, however, and an intercept at $D_{res}\approx 0$. Note that the same trend was observed in a series of natural rubber samples studied in [22].

Note also that the samples with the higher stress values, corresponding to the higher crosslink densities, exhibit strain hardening, that is, an upward deviation with respect to the Mooney–Rivlin shape, at high strain values. The strain value at onset of this non-linear hyper-elastic behavior tends to decrease as the crosslink density increases. This may suggest that this behavior is related to the deviation of network strands from Gaussian elasticity at high chain stretching, which occurs earlier as network strands are shorter. However, the failures of the various samples generally occur too early and are much too scattered to extract a significant trend in that regard.

According to rubber elasticity, the zero strain modulus should be proportional to the crosslinked density $\nu \propto 1/M_{c}$ and thus to $D_{res}$; see, e.g., Equations (4) and (16). As shown in the inset in Figure 11a, this is effectively observed (within experimental uncertainties). To further illustrate this property, the stress values were normalized by the NMR parameter $D_{res}$, assumed to be proportional to ν. The resulting normalized stress–strain curves are shown in Figure 10b. As expected, the curves superpose fairly well at small strain, over a quite large range of strain, typically up to $\lambda^{2}-\lambda^{-1}\approx 2$, which corresponds to about 60% strain. However, due to the varying Mooney–Rivlin behavior discussed above, the curves strongly deviate with respect to each other at higher strain values. These deviations correspond to the widely varying values of the ratio $C_{1}/C_{2}$.

### 4.5. Response in Terms of Segmental Orientation

**Figure 12:**
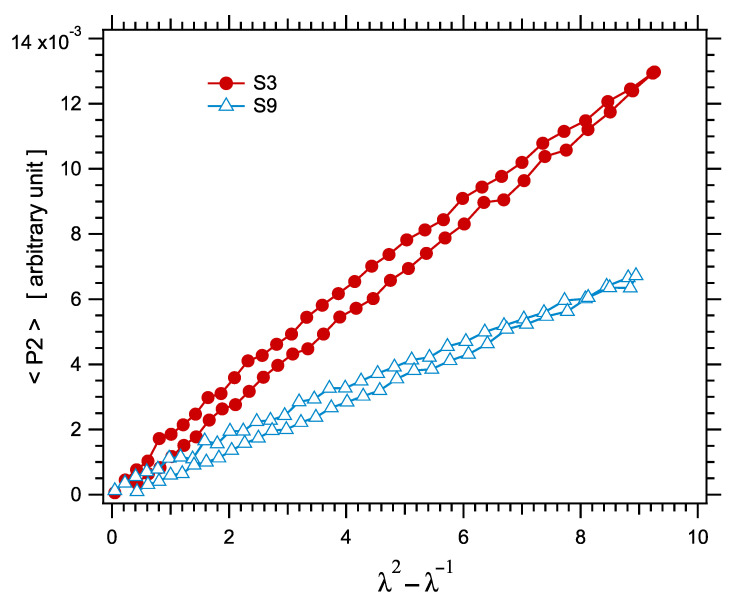
Segmental orientation parameter $P_{2}$ measured along stress–strain cycles in two representative samples S3 and S9.

The segmental orientation parameters $P_{2}$ induced upon uniaxial loading were measured in real time by X diffraction during tensile tests. According to the discussion in Section 2.4, a quantity proportional to the segmental orientation parameter introduced in Equations (2) and (3) is measured from the induced anisotropy of the wide angle scattering pattern. For the sake of simplicity, the measured parameter shall be simply denoted $P_{2}$ or $\langle P_{2}\rangle$ in what follows. In agreement with Equation (10), $P_{2}$ increases nearly linearly with the elongation parameter $\lambda^{2}-\lambda^{-1}$. This variation is nearly perfectly reversible. Two representative $P_{2}$ curves obtained during stretching cycles are shown in Figure 12.

**Figure 13:**
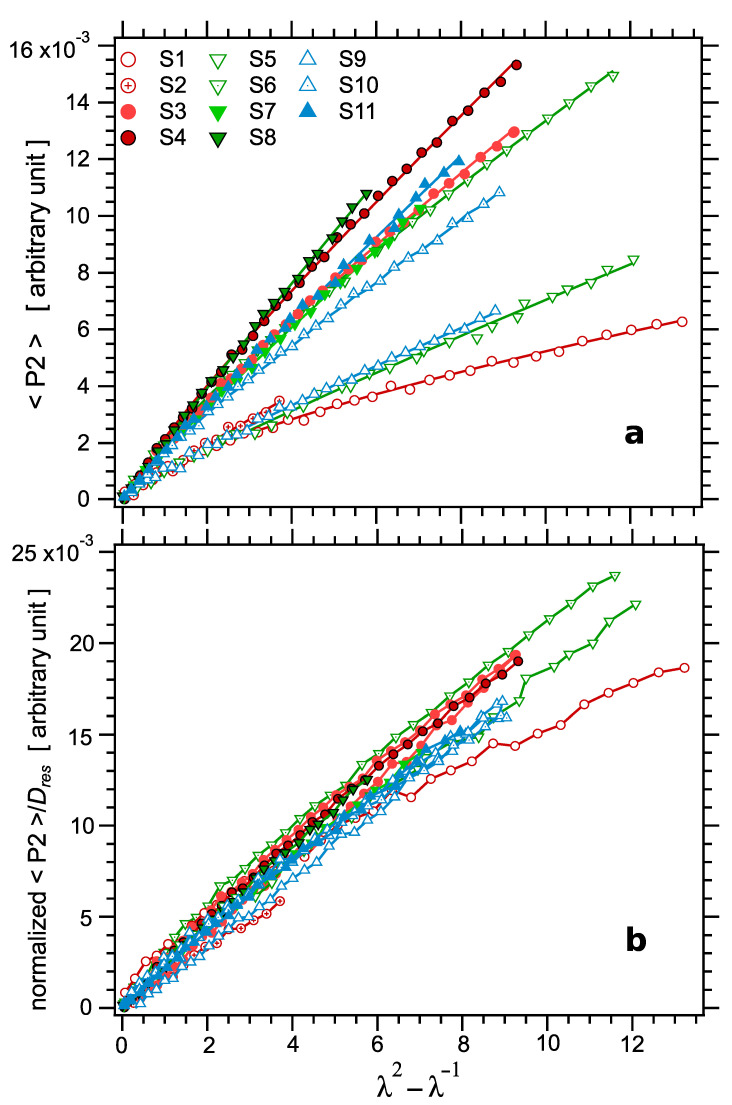
(**a**) Segmental orientation parameter $P_{2}$ measured in the series of unfilled samples, as a function of the elongation parameter $\lambda^{2}-\lambda^{-1}$. Symbols are experimental points, curves are Mooney–Rivlin fits, according to Equation (16). The graph illustrates the good fits which are obtained over the whole elongation range for all curves. (**b**) The normalized segmental orientation parameter $P_{2}/D_{res}$ as a function of the elongation parameter $\lambda^{2}-\lambda^{-1}$.

The orientation parameters $P_{2}$ are plotted in Figure 13a as a function of the elongation parameter $\lambda^{2}-\lambda^{-1}$ for the series of samples. For each value of the Acc/S ratio, the slope increases as the amount of sulfur increases, i.e., as the crosslink density increases, in qualitative agreement with the stress-optical law. To check the stress-optical law in a more quantitative way, the orientation parameter $P_{2}$ has been normalized by the NMR parameter $D_{res}$, supposed to be proportional to the crosslink density. The normalized $\langle P_{2}\rangle$ curves are plotted in Figure 13b. All curves superpose relatively well, even though the sensitivity of these measurements is lower than stress measurements.

The $<P_{2}>$ curves show a similar trend as stress–strain curves as regards the downwards inflexion at high strain related to the Mooney–Rivlin behavior, even though this trend is less pronounced for $P_{2}$ than for the stress (see, for example, the curve of sample S1). Accordingly, the curves were fitted by Equation (17), analogous to the Mooney–Rivlin model [89,90]:

(17) $$\langle P_{2}\rangle =\left(D_{1}+\frac{D_{2}}{\lambda }\right)\left(\lambda^{2}-\lambda^{-1}\right).$$

The fitted coefficients $D_{1}$ and $D_{2}$ are reported in Table 3 and plotted as a function of $D_{res}$ in Figure 11b. The coefficient $D_{1}$, which corresponds to the effective slope at large strain, shows a linear trend as a function of $D_{res}$, with a nonzero intercept of order $D_{res}^{\left(0\right)}\approx 0.31$ kHz, in reasonable agreement with the value 0.35 kHz determined before in the various independent measurements. For $D_{2}$ and the zero strain effective slope $D_{1}+D_{2}$, the data are much too scattered to confidently assess a trend.

**Figure 14:**
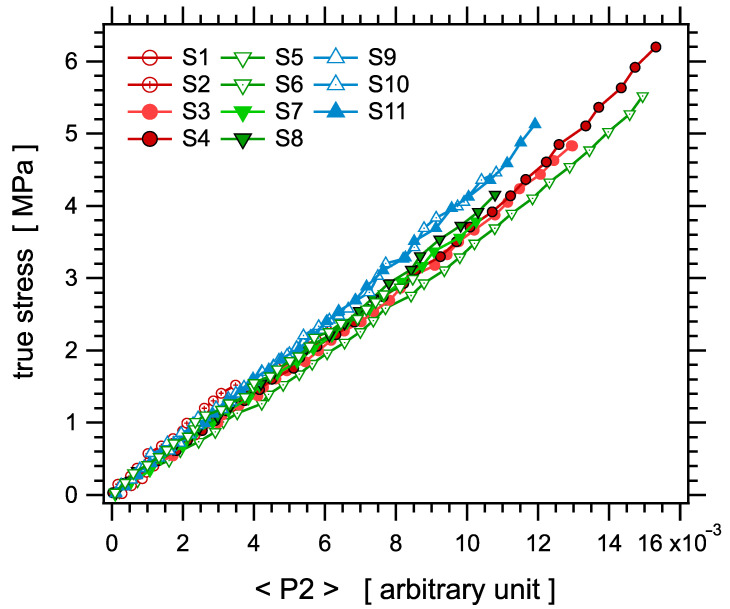
True stress as a function of the orientation parameter $P_{2}$.

Let us finally examine the stress-optical law, that is, the relationship between the stress and the orientation parameter $<P_{2}>$. According to Equation (2), the stress σ should be proportional to $<P_{2}>$ with a unique coefficient in a given elastomer matrix and at a given temperature, namely a coefficient independent of the crosslink density. Figure 14 shows the true stress σ as a function of $<P_{2}>$ in the series of samples. The linear relationship between both quantities is quite well verified over a large range of strain values, typically up to $\lambda^{2}-\lambda^{-1}\approx 12$, which corresponds to a strain of about 250% or, equivalently, up to a stress value of order 4.5 MPa. The slight upward deviations of the curve observed beyond those values come from the differences in the ratios of the Mooney–Rivlin coefficients $C_{1}/C_{2}$ and $D_{1}/D_{2}$. Within experimental uncertainties, the slopes of all curves are identical, which demonstrates the scaling expressed in Equation (2).

The proportionality factor, as denoted by *K* in Equation (10), may be estimated by considering the value of the common slope of all curves in Figure 13b. The value of the common slope $(<P_{2}>/D_{res})/(\lambda^{2}-\lambda^{-1}$) is of order $2\times 10^{-3}$, which, once substituted in Equation (10), gives Equation (18) taking ψ=1−2/f=1/2)

(18) $$\frac{<P_{2}>}{\lambda^{2}-\lambda^{-1}}\frac{1}{D_{res}}\approx \frac{K}{10N}\frac{M_{c}}{345}\approx \frac{K\rho_{r}V_{s}}{10\times 345}\approx 2\times 10^{-3},$$

where Equation (15) was used in the simplified form $D_{res}\approx 345/M_{c}$ with $M_{c}$ in g/mol and $D_{res}$ in Hz (or equivalently, $M_{c}$ in kg/mol and $D_{res}$ in kHz) and the number of segments was identified to the number of monomers ($V_{s}=106.2$ cm${}^{3}$ mol${}^{-1}$ is then taken as an average monomer volume). Equation (18) then gives −K≈0.06 (a minus sign, indicating that the scattering is reinforced perpendicular to the tensile direction, should in principle be introduced, as mentioned in Section 2.4.

**Figure 15:**
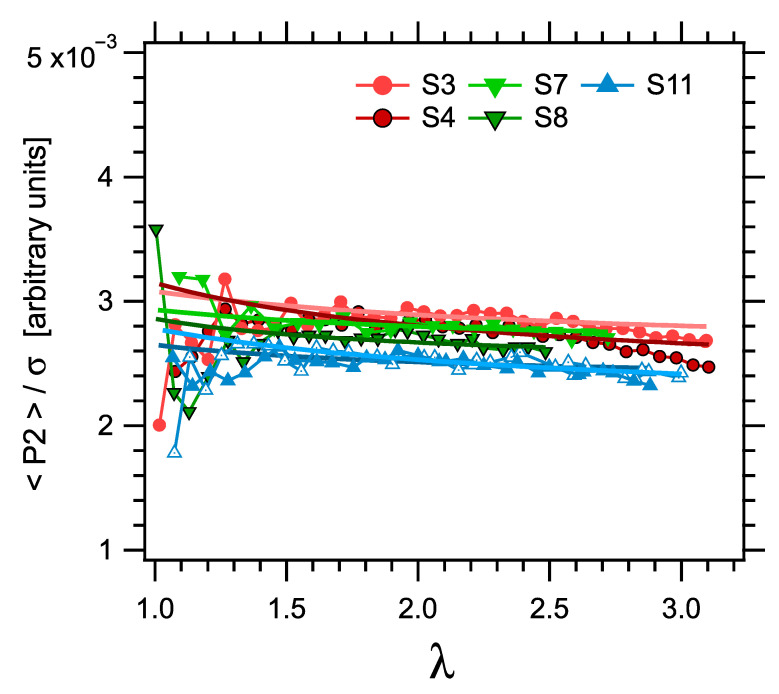
The ratio $\langle P2\rangle /\sigma$ as a function of the elongation parameter λ in a set of samples limited to those with higher apparent crosslink densities (higher $D_{res}$ values). The stress-optical law would correspond to a constant ratio. The absolute values of the ratio are arbitrary, as the anisotropy parameter $\langle P2\rangle$ deduced from X-ray scattering is proportional to the segmental order parameter, with a proportionality factor which is not known quantitatively. Symbols are measurements, curves correspond to Mooney–Rivlin fits of the stress and orientation data.

Another equivalent way of illustrating the basic relationship between σ and $\langle P_{2}\rangle$, and its validity over a large range of strain values, is to plot the ratio $\langle P_{2}\rangle /\sigma$ as a function of the elongation parameter λ, as shown in Figure 15. Note that the absolute values of the ratio are arbitrary, as the anisotropy parameter ${\langle P_{2}\rangle }_{X}$ deduced from X-ray scattering (which is, in fact, used here) is proportional to the segmental order parameter, with a proportionality coefficient estimated above but not known in a precise quantitative way. Results in a reduced set of samples limited to those with higher apparent crosslink densities (higher $D_{res}$ values) are shown as the scatter of the curves is larger in other samples. All curves shown in Figure 15 show similar trends. The ratio $\langle P_{2}\rangle /\sigma$ tends to increase (by about 20% at most) as λ increases from 1 (relaxed state) up to about 1.4 (40% strain), and then to decrease slightly with a very small slope, the relative decrease being about 4% as λ increases from about 1.4 up to about 3. This trend, and specifically the increase at lower strain values, is qualitatively similar to that observed in natural rubber [76]. Note also that points at low extension (λ close to one) are of course affected by large scattering and error bars, as these correspond to ratios between two small quantities. Curves obtained from the Mooney–Rivlin fits of both the stress and orientation data are also shown in Figure 15. These curves account for the slight decreasing trend mentioned above.

## 5. Conclusions

The observed behavior shows quite slight deviations with respect to the general stress-orientation linear relationship over a wide range of elongation ratio. These slight deviations would probably require more precise experimental investigation to be confirmed, systematized, and quantified in order for them to be compared with advanced rubber elasticity models. We would like to emphasize that the proposed approach may be applied in a wide range of materials. Specifically, orientation measurements by X-ray scattering, as presented here, are a new way of investigating the stress-optical law in materials which are not transparent in the visible light. Even though, beyond the present SBR samples, it has been applied in a limited set of materials yet, namely natural rubber and polychloroprene [22,23], the technique may potentially be applied in any elastomer material due to the intrinsically anisotropic scattering at the scale of the Kuhn segment in network chains. Industrial materials with complex formulae may be studied. One major benefit of such orientation measurements is that they give access to local strain at the scale of network chains. This is a key asset when reinforced materials are considered. In fact, it was shown that the stress-optical law, Equation (2), is no longer verified in elastomers reinforced by nanometric silica aggregates [24,25], as the stress in reinforced materials no longer follows the same variation as the orientation parameter in the elastomer matrix. It was argued that such investigations in reinforced materials may enable discriminating reinforcement mechanisms, issuing from the response of the matrix (strain amplification) and those from the filler network. However, to obtain such a quantitative comparison, the behavior of the matrix needs to be quantitatively assessed in detail, which is the topic of the present paper. Note finally that a key aspect regarding rubber elasticity, the stress-optical law, and the behavior of reinforced elastomer is the effect of temperature. Based on rubber elasticity theory, the average chain orientation parameter $\langle P_{2}\rangle$ should be independent of temperature in elastomers, which has been demonstrated long ago [79]. This was shown to be true even in reinforced materials [25]. By contrast, one specific, emerging behavior in reinforced materials is indeed the temperature variation of the mechanical response [10,25]. This temperature variation has been interpreted as indirect proof that reinforcement mechanisms different from mere strain amplification in the matrix occur in materials reinforced with nanoparticles.

## Figures and Tables

**Table 1:** List of the SBR samples. CBS and DPG are accelerators. All amounts are expressed in g and are relative to 137.5 g of oil-extended SBR.

Samples	Sulfur (g)	CBS (g)	DPG (g)	Ratio Acc/S	Ratio CBS/S
S0	0	0	0	0	
S1	0.4	0.4	0.3	1.75	1
S2	1.1	1.1	0.83	1.75	1
S2b	1.1	1.1	0.83	1.75	1
S3	1.6	1.6	1.2	1.75	1
S4	2.2	2.2	1.65	1.75	1
S5	0.4	0.73	0.55	3.18	1.82
S6	1.1	2	1.5	3.18	1.82
S7	1.6	2.91	2.18	3.18	1.82
S8	2.2	4	3	3.18	1.82
S9	0.4	1	0.75	4.38	2.5
S10	1.1	2.8	2.06	4.38	2.5
S11	1.6	4	3	4.38	2.5
S12	2.2	5.5	4.13	4.38	2.5

**Table 2:** NMR residual dipolar coupling $D_{res}$ (as determined from Equation (13)), equilibrium swelling ratio *Q* (ratio of swollen gel over polymer volumes expressed in %), and torque difference ΔΓ from rheometry measurements in the set of samples. The indicated inverse molecular mass values $1/M_{c}$ values are from swelling measurements, using parameter values indicated in the text.

Samples	$D_{\mathrm{res}}$	*Q*	ΔΓ	$1/M_{c}$
	(kHz)	(%)	(dN·m)	(mol/g)
S0	0.340	-	-	-
S1	0.422	896	9.8	0.000110
S2	0.564	426	24	0.000526
S2b	0.585	391	25.7	0.000526
S3	0.680	364	30.4	0.000756
S4	0.793	319	35	0.00104
S5	0.454	711	12	0.000175
S6	0.638	376	27.5	0.000701
S7	0.737	331	34.3	0.000947
S8	0.867	286	38.5	0.00136
S9	0.461	609	14.4	0.000241
S10	0.665	353	31	0.000813
S11	0.758	311	35.4	0.00110
S12	0.859	276	39.8	0.00148

**Table 3:** Mooney–Rivlin parameters $C_{1}$ and $C_{2}$ obtained by fitting stress–strain curves in Figure 10 with Equation (16). Effective Mooney–Rivlin parameters $D_{1}$ and $D_{2}$ obtained by fitting the average orientation parameter curves in Figure 13 with Equation (17). See also Figure 11.

Samples	$C_{1}$	$C_{2}$	$D_{1}$	$D_{2}$
	(MPa)	(MPa)		
S1	0.0656	0.414	0.000165	0.00115
S2	0.254	0.319	0.000774	0.000316
S3	0.406	0.305	0.00104	0.00115
S4	0.555	0.224	0.00129	0.00116
S5	0.157	0.313	0.000551	0.000493
S6	0.406	0.218	0.000865	0.00151
S7	0.463	0.171	0.00121	0.00065
S8	0.693	0.0482	0.00171	0.000409
S9	0.172	0.353	0.000552	0.000592
S10	0.414	0.2681	0.000877	0.00102
S11	0.5683	0.135	0.00133	0.000534
